# Removal of cadmium from contaminated *Lentinula edodes* by optimized complexation and coagulation

**DOI:** 10.1002/fsn3.384

**Published:** 2016-05-31

**Authors:** Yi Wang, Chen Wang, Wei Cheng, Yinbing Bian, Peng Guo

**Affiliations:** ^1^ Hubei Academy of Agricultural SciencesInstitute of Agricultural Products Processing and Nuclear Agriculture Technology ResearchWuhan430064China; ^2^ Institute of Applied MycologyHuazhong Agricultural UniversityWuhan430070China

**Keywords:** Cadmium, heavy metal pollution, lentinan, *Lentinula edodes*

## Abstract

Heavy metal pollution is a serious problem for *Lentinula edodes*; however, the treatment of contaminated *L. edodes* has seldom been studied. This study investigated the removal of cadmium (Cd) from contaminated *L. edodes* and its lentinan by complexation and coagulation. Some influencing factors, such as pH, medical dosage, and preoxidation were examined. Cd complexation from contaminated *L*. *edodes* was shown to be more efficient under acidic conditions (pH 5.0), with a clearance rate of 80.47% in 25 mmol/L EDTA and 78.45% in 25 mmol/L sodium citrate. The Cd content in the lentinan of the contaminated *L. edodes* was markedly lower than that in the powdered mushroom (2.77 mg/kg vs. 19.49 mg/kg) and was easier to remove. The maximum Cd clearance rate (96.3%) for lentinan was obtained using an optimized process that involved preoxidation with 0.5 mg/L KMnO_4_, complexing with 25 mmol/L EDTA and 25 mmol/L sodium citrate, and coagulation with 50 mg/L activated carbon (AC) at pH 10.0.

## Introduction

Recently, with rapid industrial development, heavy metal pollution has become an increasing problem, leading to serious food security risks (Campanella et al. 1994). *Lentinula edodes*, commonly known as the shiitake mushroom, is one of the most popular edible mushrooms and the second most cultivated mushroom in the world (Breene 1990). It is highly nutritious with a characteristic fragrance and important medical value (Bisen et al. 2010). Heavy metal pollution is an even more serious problem in *L. edodes* because the mushroom tends to accumulate heavy metals (Melgar et al. 1998; Thomet et al. 1999; Demirbas 2001). It has been documented that *L. edodes* and other saprophytic mushrooms can be used to remove heavy metals efficiently (Gabriel et al. 1996; Melgar et al. 2007; Chen et al. 2008), which reflects the strong heavy metal sorption capability of *L. edodes*. Thus, purifying heavy metal–contaminated *L*. *edodes* would be a challenging endeavor.

Cadmium (Cd) is one of most notoriously toxic heavy metals and is included in the Environmental Protection Agency's list of priority pollutants (Cameron 1992). It combines with sulfhydryl groups in proteins and inhibits enzyme activity, which is detrimental to humans (Thomet et al. 1999). The famous itai‐itai disease in Toyama Prefecture was caused by Cd poisoning (Murata et al. 1971). One recent investigation indicated that over 50% of the Cd content of the *L. edodes* cultivated in Hubei province of China exceeded the limit (Yang et al. 2014).

Fungal interactions with toxic heavy metals have been investigated previously. It was speculated that accumulation of heavy metals by mushrooms involves two processes. One possible mechanism is the bioaccumulation process in which essential metal ions are substituted by heavy metals (Brunnert and Zadrazil 1985). The other mechanism is biosorption, such as adsorption, ion exchange processes, and complexation with functional groups (Płaza et al. 1996; Mar'in et al. 1997). It has been proposed that one possible way of removing the heavy metals may be to release them through competitive binding by other strong metal‐binding agents.

Various methods, such as chemical precipitation (Ku and Jung 2001), ion exchange (Alyüz and Veli 2009), membrane filtration (Landaburu‐Aguirre et al. 2009), and adsorption and coagulation using polymer materials (Dias et al. 2007) have been employed for the removal of heavy metal contamination. Complexing agents, such as EDTA (Peters 1999; Zou et al. 2009) and sodium citrate (Gao et al. 2003; Yuan et al. 2007), have been used to leach heavy metals from contaminated soil, and have also been used as food additives. However, many methods are not suitable for purifying contaminated *L*. *edodes*, for food safety reasons.

Another restriction is the insolubility of the mushroom, which limits the use of an identical insolubility adsorbing material and coagulating agents. Moreover, the complex composition of *L.  edodes* makes heavy metal removal challenging. Lentinan is one of the most important active constituent of *L. edodes*, which has many biological effects, such as antiviral, immune regulatory, and antitumor effects, etc. (Sasaki and Takasuka 1976; Zhang et al. 2011).

The aim of this study was to explore processes for removing toxic heavy metal (Cd) from polluted *L. edodes*. Complexing agents (EDTA and sodium citrate) were used to leach heavy metal from contaminated *L. edodes*. Lentinan was then extracted, and complexing and coagulating agents (active carbon, polyaluminium chloride, and chitosan) were applied to remove the heavy metal. Some factors that can affect the binding capacity, such as pH, the dosage of agents used, and the initial valence state of Cd (by addition of oxidant), were investigated in the course of optimizing this process. This work filled the gaps in the study of dealing with heavy metal–polluted mushroom, with regard to potential use in treating of heavy metal–polluted food or medicinal sources.

## Materials and Methods

### Assays of Cd accumulation by L. edodes

*L. edodes* (L26) was obtained from the Institute of Applied Mycology, Huazhong Agricultural University, Wuhan, China. It was maintained on a medium containing malt extract (20 g/L), yeast extract (1 g/L), peptone (1 g/L), and glucose (20 g/L). The cultivation substrate contained 80% sawdust, 19% wheat bran, 1% gypsum (CaSO4·2H_2_O), and had a water content of about 60%. Cd(NO_3_)_2_ was added to the cultivation substrate to a final Cd concentration of 0, 0.5, 1, 2, 2.5, 5, 7.5, or 10 (mg/kg dry weight). Twelve replicates were performed for each concentration. Once grown, the mushroom cap was collected for further investigations. Cd contents in the compost and the cap were examined to calculate the Cd enrichment factor, as described in “Determination of Cd concentration”.

### Determination of Cd concentration

Cd concentrations were determined by atomic absorption spectrometry (AAS) after dry‐ashing pretreatment, using a carbon rod atomizer (Gast et al. 1988). Duplicates of *L*. *edodes* samples were prepared independently.

### Treatment of polluted L. edodes to remove Cd

Cd‐polluted *L. edodes* was ground in a high‐speed disintegrator to a particle size of 800 *μ*m and dried to a constant weight at 55°C. Two grams of *L. edodes* powder was leached using 50 mL deionized water containing disodium‐EDTA or sodium citrate at various concentrations, as indicated. After 1 h incubation at 25°C on a rotary shaker at 100 rpm, the mixtures were centrifuged at 12000 g for 10 min. The precipitates were collected, and the Cd concentration was determined after drying to a constant weight. Each sample was prepared in triplicate. The effect of pH was investigated by using different volumes of acid (1 mol/L HCl) or alkali (1 mol/L NaOH) to adjust the pH of the mixture.

### Extraction of lentinan

Dried *L. edodes* powder was extracted with deionized hot water (powder:water ratio of 1:25 [v/v]) at 120°C and 0.1 Mpa for 1 h. After centrifuging at 12000 g for 10 min, the aqueous phase was collected and concentrated to about half the initial volume under reduced pressure. Four volumes of precooled 95% ethanol were added to the solution and the mixture was stored overnight at 4°C. The precipitates were collected by centrifugation at 12000 g for 10 min, and then washed with 75% ethanol. The crude lentinan obtained in this way was dried to a constant weight at 55°C (Ye and Jiang 2011).

### Treatment of the lentinan to remove Cd

Complexation and coagulation were carried out to remove Cd in the lentinan prior to ethanol precipitation. For complexation, various concentrations of disodium EDTA or sodium citrate were added to the aqueous phase containing the lentinan described in “Extraction of lentinan”. After a 1‐h incubation at 25°C while shaking at 100 rpm, followed by 20 min centrifugation at 12000 g, the supernatant was collected, ethanol precipitated, and later process was performed as described in “*Extraction of lentinan*”. For coagulation, various amounts of activated carbon (AC), polyaluminium chloride (PAC), or chitosan was added to the aqueous phase containing lentinan. After incubation for 1‐h incubation at 25°C while shaking at 100 rpm, the coagulation precipitate was removed by 20 min centrifugation at 12000 g, and ethanol precipitation was performed subsequently as described in “*Extraction of lentinan*”. The effect of pH, by using 1 mol/L HCl or 1 mol/L NaOH, and the concentrations of the complexation or coagulation agents were investigated.

### Preoxidation by KMnO_4_

The effect of preoxidation was investigated by adding 0.5 mg/L KMnO_4_ to the aqueous phase described in “Extraction of lentinan”, prior to the complexation and coagulation process. Preoxidation was performed at 25°C, for 30 min while shaking at 100 rpm.

## Results and Discussion

### Cd accumulation by L. edodes

**Figure 1:**
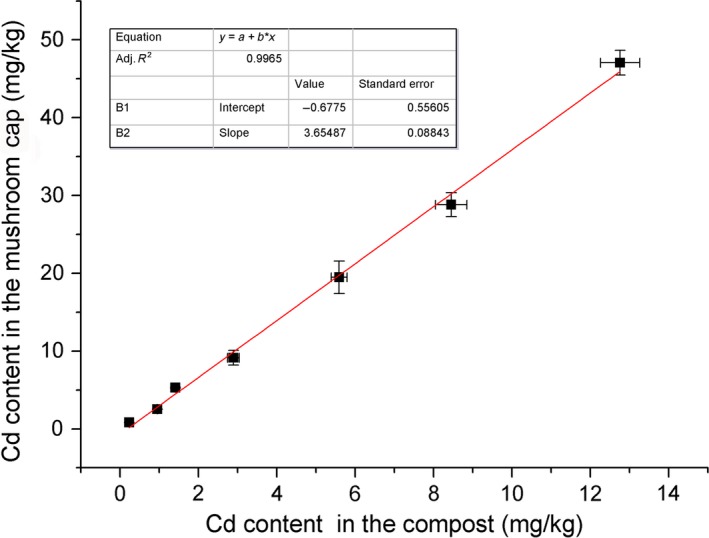
Cadmium (Cd) accumulation by *Lentinula edodes*. Cd was added to the mushroom cultivation substrate to a theoretical value of 0, 0.5, 1, 2, 2.5, 5, 7.5, or 10 mg/kg dry weight. The correlation between the actual Cd content in the compost and the mushroom cap was determined, and it corresponded to the formula *y* = 3.655*x*−0.678 (*R*² = 0.997). Bars represent the standard deviation of 12 replicates.

*L. edodes* has been reported to accumulate heavy metals from the environment (Holan and Volesky 1995; Demirbas 2001). Here, we deliberately contaminated *L. edodes* with Cd by adding Cd to the compost, and investigated the enrichment factor. Figure 1 shows that the Cd content in the *L. edodes* cap and in the compost was significantly positively correlated, corresponding to the formula *y* = 3.655*x*−0.678 (*R*² = 0.997), in which x was the practical Cd content in the compost and y was the Cd content in the *L. edodes* cap (Fig. 1). This indicated that *L. edodes* had a strong capacity for Cd accumulation. A sample containing 19.49 mg/kg Cd was used in the later Cd removal experiment.

### Removal of Cd from polluted L. edodes by complexation

**Figure 2:**
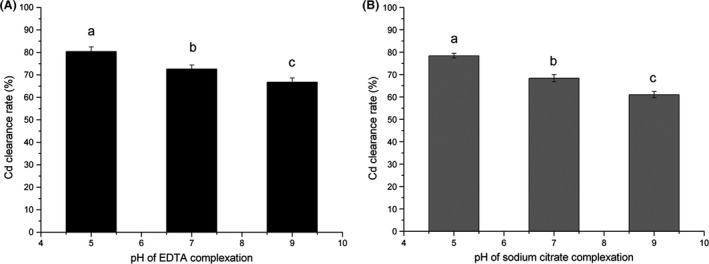
Effect of pH on cadmium (Cd) removal from polluted *Lentinula edodes* by complexation. A solution of 10 mmol/L EDTA (A) or 10 mmol/L sodium citrate (B) was used, and the effect of pH effect on them was detected independently. Bars represent the standard deviation of the set of values. Values (means of three replicates) not sharing common letters are significantly different (one‐way ANOVA with the Student‐Newman‐Keuls method, *P* < 0.05).

EDTA and sodium citrate are known to be efficient chelating agents that can form strong metal‐ligand complexes, and they are widely used in restoring heavy metal–contaminated soil (Peters 1999; Gao et al. 2003). In this study, 10 mmol/L EDTA or sodium citrate was used to complex Cd from contaminated mushrooms. As shown in Figure 2, the Cd clearance rate (denoted as the ratio of Cd content of the treated sample to the initial Cd content in the contaminated mushrooms) at pH 7.0 was 72.65% and 68.41% for EDTA and sodium citrate, respectively (Fig. 2).

As it is known that pH is a very important factor affected complexing (EDTA, e.g., EDTA^4−^ + nH^+^↔H_n_EDTA^(n−4)+^[*n* = 1, 2, 3, 4]; EDTA^4−^ + nH^+^ + Cd^2+^ ↔ CdH_n_EDTA^(n−2)+^[*n* = 0,1]). Moreover, Cd(OH)_2_ precipitation occurred in an alkaline solution (Cd^2+^ +2OH^−^ ↔ Cd(OH)_2_), which might be adsorbed by the mushroom biomass and may obstruct Cd removal (Basta and Tabatabai 1992; Nastaran et al. 2011). When the effect of pH was examined, including an acidic condition at pH 5.0, a neutral condition at pH 7.0, and an alkaline condition at pH 9.0, respectively, Cd was found to be more easily removed under acidic conditions. At pH 5.0, the Cd clearance rate was the highest; that is, 80.47% in EDTA and 78.45% in sodium citrate. This complemented the report of Melgar and others who found that mushroom biomass could absorb most heavy metal from a solution at an alkaline pH (Melgar et al. 2007).

### Extraction of lentinan

So far, we had shown that EDTA and sodium citrate could remove a fair amount of Cd from contaminated mushrooms; however, approximately 20% of the Cd remained, which might exceed the safety limit under heavily polluted conditions. Thus, a more efficient process was needed. Nevertheless, the insolubility of the mushroom powder excluded the use of many insolubility additives that are typically used to remove heavy metals. Moreover, the complex physical structure and multiplicity of functional groups in the mushroom components are the main reason for its heavy metal accumulation (Volesky 2007). Therefore, we speculated that preparing simple soluble components might allow us to address this issue. As lentinan is the most important active constituent of this mushroom, we chose it as a candidate component.

Crude lentinan was extracted as described in “Extraction of lentinan”. The Cd content in the lentinan extract was markedly less than that in the mushroom per se (19.49 mg/kg vs. 2.77 mg/kg; data not shown). This indicated that Cd adsorption capacity of the lentinan was lower than that of the mushroom, which might be due to its simple physical structure and low number of functional groups. We then attempted to remove Cd from the lentinan in subsequent experiments.

### Cd removal of lentinan by complexation

**Figure 3:**
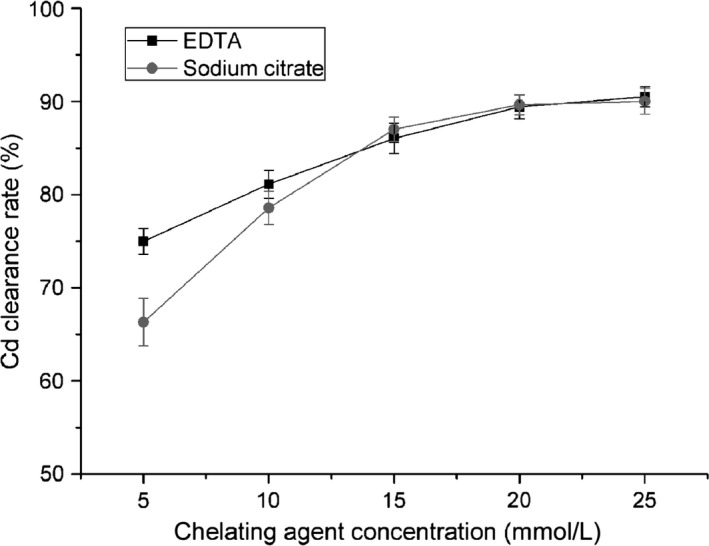
Effect of dosage on cadmium (Cd) removal from lentinan by complexation. The effect of the concentration of EDTA or sodium citrate was examined independently using a concentration series (5, 10, 15, 20, and 25 mmol/L). Means of three replicates are presented, and bars represent the standard deviation of the set of values.

The chelating agents, 10 mmol/L EDTA or 10 mmol/L sodium citrate, were applied individually during lentinan extraction at pH 7.0, and the Cd‐chelate complex would remain in the aqueous phase and could be removed from the lentinan after ethanol precipitation. The Cd clearance rate was denoted as the ratio of the Cd content of the treated lentinan to the Cd content of untreated lentinan. We showed that the Cd clearance with 10 mmol/L EDTA and 10 mmol/L sodium citrate in lentinan was 81.10% and 78.58%, respectively (Fig. 3), which was even higher than that in the mushroom powder. This was probably due to the weaker Cd adsorption capacity of lentinan. The mechanism of heavy metal removal was mainly via competitive binding; therefore, it was easier for the chelating agents to compete with lentinan than with the mushroom powder.

#### Effect of dosage

The effect of the concentration of EDTA and sodium citrate (5, 10, 15, 20, and 25 mmol/L) was examined at pH 7.0. For EDTA, the Cd clearance rate was 74.96% at 5 mmol/L, and clearance was positively correlated with the EDTA concentration. The maximum clearance rate of 90.49% was obtained at an EDTA concentration of 25 mmol/L (Fig. 3). Similar findings were achieved for sodium citrate. At 5 mmol/L sodium citrate, the Cd clearance rate was 66.31%, and this increased as the sodium citrate concentration increased. The maximum clearance rate of 90.01% was obtained at 20 mmol/L sodium citrate (Fig. 3).

#### Effect of pH

**Figure 4:**
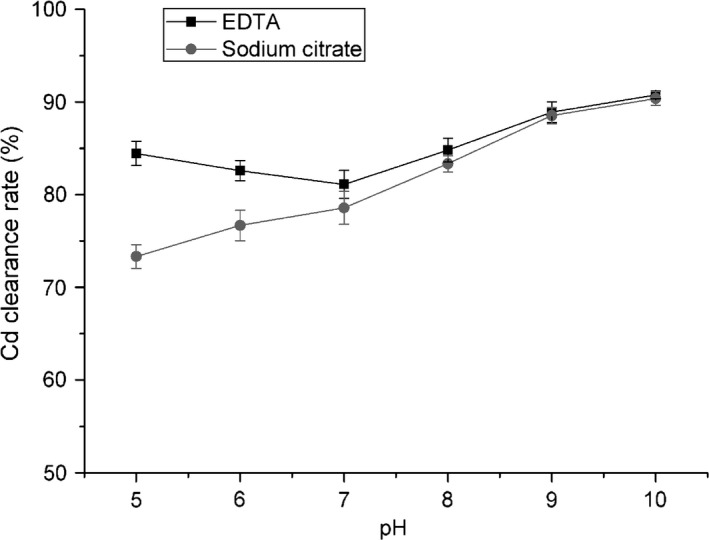
Effect of pH on cadmium (Cd) removal from lentinan by complexation. The effect of the pH of EDTA or sodium citrate solution was examined independently using a pH series (pH 5, 6, 7, 8, 9, and 10). Means of three replicates are presented, and bars represent the standard deviation of the set of values.

As previously described, pH is a crucial factor that affects the complexing between Cd and chelating agents (Nastaran et al. 2011). The effect of pH was examined individually when various additives were used. As shown in Figure 4, the Cd clearance rate slightly decreased as pH increased from 5 to 7. Zou et al. had studied the pH effect when using EDTA to leach heavy metal from polluted soil, and found that the efficiency of heavy metal removal decreased as pH increased (Zou et al. 2009). Moreover, it has been reported that the heavy metal removal efficiency follows the order of H_4_‐EDTA > Na_2_EDTA > Na_4_EDTA (Papassiopi et al. 1999). Thus, it was reasonable that the Cd clearance would decrease from pH 5 to pH 7. However, when the pH was higher than 7, the removal of Cd showed a marked increment. It is known that Cd tends to form a hydroxide, Cd(OH)_2_, at pH >7 (Basta and Tabatabai 1992). During soil washing, Cd(OH)_2_ precipitates would be absorbed and will not be removed; however, in our study, lentinan was present in the aqueous phase. Thus, Cd(OH)_2_ precipitation actually facilitated Cd removal, so that Cd clearance would increase when pH >7. Moreover, the polysaccharide structure is unstable under alkaline conditions (Cintra and Takagi 2015), which may destabilize its binding to Cd. Additionally, it was noted that excessively high alkaline pH should be avoided so as not to damage the lentinan.

The Cd clearance rate of sodium citrate increased with increasing pH in the pH 5–pH 10 range. Other than the effects of Cd(OH)_2_ precipitation and lentinan stability, these results also corresponded to previous studies on heavy metal chelation by citrate. Citrate (denoted as H_3_L) changes from H_3_L, H_2_L^−^, and HL^2−^, to L^3−^ as the pH increases. HL^2−^ and L^3−^ show greater chelating ability for heavy metals than do H_3_L and H_2_L^−^ (Martell and Smith 1977). Thus, the Cd clearance under alkaline conditions in the presence of HL^2−^ and L^3−^ was greater than that under acid conditions in the presence of H_3_L and H_2_L^−^.

### Cd removal from lentinan by coagulation

**Figure 5:**
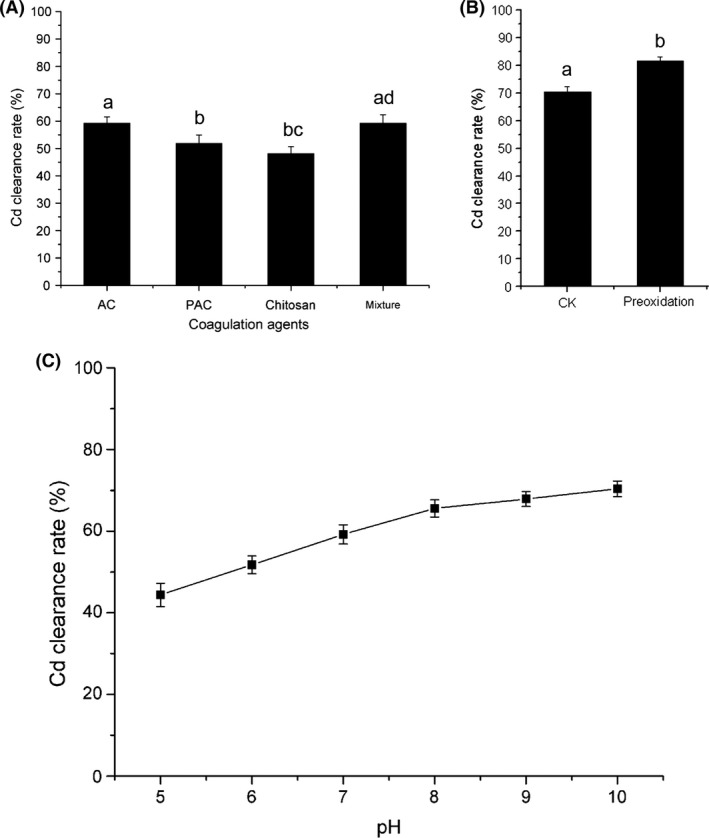
Cadmium (Cd) removal from lentinan by coagulation. **(**A) Use of different coagulation agents; (B) Effect of different pH (pH 5, 6, 7, 8, 9, and 10); (C) Effect of preoxidizing with 5 mg/L KMnO_4_. Means of three replicates are presented. Bars represent the standard deviation of the set of values. Values (means of three replicates) not sharing common letters are significantly different (one‐way ANOVA with Student‐Newman‐Keuls method, *P* < 0.05).

Activated carbon was used as adsorbent because of its large surface area (Jusoh et al. 2007). PAC is a commercial coagulant widely used in wastewater treatment (El‐Sikaily et al. 2007). Chitosan is a natural polymer used as a new type of heavy metal flocculant (Bratskaya et al. 2009). These agents were added individually to the lentinan aqueous phase before ethanol precipitation, at pH 7.0. In the process, Cd would be absorbed and coagulated, and then removed by centrifugation. The Cd clearance rates with 90 mg/L activated carbon, 90 mg/L PAC, and 90 mg/L chitosan were 59.21%, 51.87%, and 48.13%, respectively (Fig. 5A). Coagulation agents could thus remove Cd from lentinan. No significant difference was detected between PAC and chitosan (*P* = 0.142), whereas AC showed the strongest competitive force among these agents. A mixture of AC, PAC, and chitosan showed a Cd clearance rate of 59.3%, which was not significantly different from that achieved by AC alone (*P* = 1.000). This implied that the mechanisms of action of these agents are likely to be similar and not cumulative. Thus, AC was used as the only coagulating agent in later experiments.

The effect of the concentration of AC was investigated using a concentration series (50, 70, 90, 110, and 130 mg/L); however, the Cd clearance rate was not significantly different (data not shown), which suggested that 50 mg/L was already a saturated concentration of AC for Cd coagulation. We did not investigate concentrations of less than 50 mg/L. The influence of pH on the Cd removing efficiency of AC was also examined. The Cd clearance rate of AC was positively correlated with the pH value (Fig. 5B). Under alkaline conditions, Cd(OH)_2_ precipitates were absorbed by the AC and facilitated the coagulation process.

Preoxidation is one of the principal methods for improving the coagulation process by destroying the organic coating on the surface of particles. KMnO_4_ preoxidation was performed prior to coagulation. Figure 5C shows that preoxidation with KMnO_4_ greatly improved Cd removal by coagulation (from 71.40% to 81.59%). Ma and Li reported that preoxidation is particularly effective for enhancing the coagulation of waste waters with a relatively high organic content (Ma and Li 1993), such as the lentinan solution used here.

However, it was noted that the Cd clearance with AC reached only 81.59% under these optimal conditions. Chang and Gang 2007 also considered that coagulation cannot treat the heavy metal wastewater completely. Therefore, a combination of coagulation and complexation would be a possible way around this problem.

### Optimization of lentinan Cd removal

**Figure 6:**
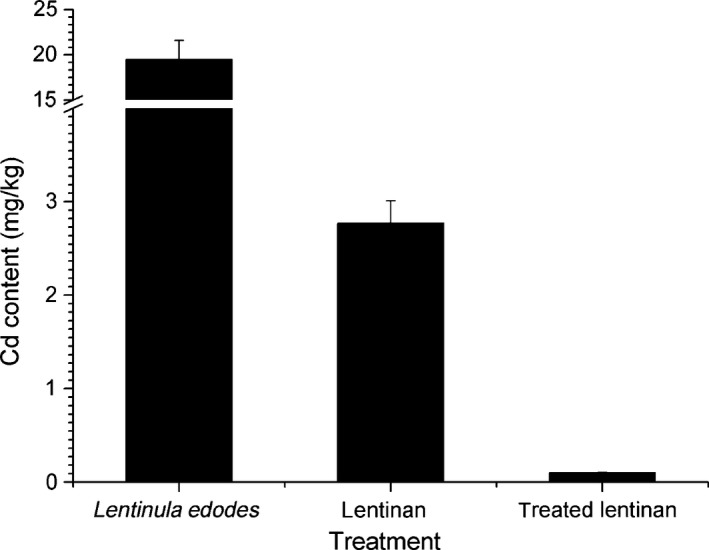
Optimization of lentinan Cd removal means of three replicates are presented. Bars represent the standard deviation of the set of values. Values (means of three replicates) not sharing common letters are significantly different (one‐way ANOVA with Student‐Newman‐Keuls method, *P* < 0.05).

After KMnO_4_ preoxidation, a combination of chelating agents (25 mmol/L EDTA and 25 mmol/L sodium citrate) and coagulation agents (50 mg/L AC) were used at pH 10.0. The final Cd content in the treated lentinan was 0.10 mg/kg, which was 3.7% of that in the untreated lentinan (2.77 mg/kg) and only 0.5% of that in the initial contaminated mushroom powder (19.49 mg/kg; Fig. 6). The optimized Cd removal process was thus proven to be very efficient.

## Conclusions

Here, we implemented new strategies to treat Cd‐contaminated *L. edodes*. Complexing agents (EDTA and sodium citrate) were used to leach the metal from the contaminated *L. edodes*. The Cd clearance rate was higher under alkaline conditions, and EDTA and sodium citrate were considered competent for general removal of Cd from contaminated *L*. *edodes* when the Cd concentration was not excessive. For heavily polluted *L. edodes*, extraction of purified lentinan would be a superior approach. Complexing agents (EDTA and sodium citrate) and coagulating agents (AC, PAC, and chitosan) were applied to remove the residual Cd from the lentinan. The Cd extraction efficiency increased with increasing pH in the pH 5–pH 10 range for sodium citrate and all three coagulating agents, whereas it decreased slightly over the range of pH 5–pH 7 and then increased from pH 7–pH 10. In general, an alkaline condition (pH 10) was optimal for Cd removal. Preoxidation with KMnO_4_ could enhance Cd removal by coagulation. Taking all these findings into consideration, an optimized process was developed for removal of Cd from lentinan, which involved preoxidizing with 0.5 mg/L KMnO_4_, complexing with 25 mmol/L EDTA and 25 mmol/L sodium citrate, and coagulating with 50 mg/L AC at pH 10.0. The optimized process obtained a Cd clearance rate of 96.3% for the lentinan, and the final Cd content of the treated lentinan was only 0.5% of that in the initial contaminated mushroom (0.10 vs. 19.49 mg/kg). This new strategy for obtaining purified lentinan from contaminated *L. edodes* was demonstrated to be effective, and also indicated that it is advisable to extract purified active ingredients from polluted materials. This study provides a new approach for the treatment of heavy metal–polluted food or medicinal sources.

## Conflict of Interest

None declared.

## Supporting information


**Table S1.** Cd content corresponding to the figure 1
**Table S2.** Cd content corresponding to the figure 2
**Table S3.** Cd content corresponding to the figure 3
**Table S4.** Cd content corresponding to the figure 4
**Table S5.** Cd content corresponding to the figure 5
**Table S6.** Cd content corresponding to the figure 6
