# Supplementary material for: Removal of cadmium from contaminated Lentinula edodes by optimized complexation and coagulation
Source: Food Sci Nutr. 2016 May 31;5(2):215–22. doi: 10.1002/fsn3.384 (PMC5332263; doi:10.1002/fsn3.384)
Supplement: Supplementary file 1 — Table S1. Cd content corresponding to the figure 1 Table S2. Cd content corresponding to the figure 2 Table S3. Cd content corresponding to the figure 3 Table S4. Cd content corresponding to the figure 4 Table S5. Cd content corresponding to the figure 5 Table S6. Cd content corresponding to the figure 6 [file FSN3-5-215-s001.doc]

**Table S1 Cd content corresponding to the figure 1**

| Actual Cd content in figure 1 (mg/kg) | | |
| --- | --- | --- |
| theoretical added content | actual content in the compost | Cd content in the cap |
| 0 | 0.23 | 0.85a* |
| 0.5 | 0.95 | 2.54 b |
| 1 | 1.41 | 5.31c |
| 2.5 | 2.89 | 9.14 d |
| 5 | 5.59 | 19.49e |
| 7.5 | 8.45 | 28.82 f |
| 10 | 12.76 | 47.07 g |

*Values (means of 12 replicates) not sharing common letters are significantly different (one-way ANOVA with the Student−Newman−Keuls method, P < 0.05)

**Table S2 Cd content corresponding to the figure 2**

| Actual Cd content in figure 2 (mg/kg) | |
| --- | --- |
| Treatment of the mushroom | Cd content |
| 10 mM EDTA (pH 5) | 3.81a* |
| 10 mM EDTA (pH 7) | 5.33b |
| 10 mM EDTA (pH 9) | 6.47c |
| 10 mM sodium citrate (pH 5) | 4.20ad** |
| 10 mM sodium citrate (pH 7) | 6.16ce*** |
| 10 mM sodium citrate (pH 9) | 7.59f |

* Values (means of three replicates) not sharing common letters are significantly different (one-way ANOVA with the Student−Newman−Keuls method, P < 0.05)

** EDTA-pH 5vs. sodium citrate pH 5, P = 0.158

*** EDTA-pH 9 vs. sodium citrate pH 7, P = 0.235

**Table S3 Cd content corresponding to the figure 3**

| Actual Cd content in figure 3 (mg/kg) | |
| --- | --- |
| Treatment of the lentinan | Cd content |
| 5 mM EDTA (pH 7) | 0.69a* |
| 10 mM EDTA (pH 7) | 0.52b |
| 15 mM EDTA (pH 7) | 0.39c |
| 20 mM EDTA (pH 7) | 0.29d |
| 25 mM EDTA (pH 7) | 0.26de** |
| 5 mM sodium citrate (pH 7) | 0.93a |
| 10 mM sodium citrate (pH 7) | 0.59b |
| 15 mM sodium citrate (pH 7) | 0.36c |
| 20 mM sodium citrate (pH 7) | 0.29cd |
| 25 mM sodium citrate (pH 7) | 0.28cde*** |

* Cd content in the lentinan treated by EDTA or sodium citrate was analyzed independently. Values (means of three replicates) not sharing common letters are significantly different (one-way ANOVA with the Student−Newman−Keuls method, P < 0.05)

** 20 mM EDTA vs. 25 mM EDTA, P = 0.361

*** 15 mM sodium citrate 20 mM sodium citrate, P = 0.086; 15 mM sodium citrate 25 mM sodium citrate, P = 0.127; 20 mM sodium citrate 25 mM sodium citrate, P = 0.801.

**Table S4 Cd content corresponding to the figure 4**

| Actual Cd content in figure 4 (mg/kg) | |
| --- | --- |
| Treatment of the lentinan | Cd content |
| pH 5 (10 mM EDTA) | 0.43a* |
| pH 6 (10 mM EDTA) | 0.48b |
| pH 7 (10 mM EDTA) | 0.53bc** |
| pH 8 (10 mM EDTA) | 0.42ad*** |
| pH 9 (10 mM EDTA) | 0.31e |
| pH 10 (10 mM EDTA) | 0.26ef**** |
| pH 5 (10 mM sodium citrate) | 0.74a |
| pH 6 (10 mM sodium citrate) | 0.65b |
| pH 7 (10 mM sodium citrate) | 0.58c |
| pH 8 (10 mM sodium citrate) | 0.46d |
| pH 9 (10 mM sodium citrate) | 0.32e |
| pH 9 (10 mM sodium citrate) | 0.27ef***** |

* Cd content in the lentinan treated by EDTA or sodium citrate was analyzed independently. Values (means of three replicates) not sharing common letters are significantly different (one-way ANOVA with the Student−Newman−Keuls method, P < 0.05)

** EDTA pH 6 vs. EDTA pH 7, P = 0.143

*** EDTA pH 5 vs. EDTA pH 8, P = 0.711

**** EDTA pH 9 vs. EDTA pH 10, P = 0.076

***** sodium citrate pH 9 vs. sodium citrate pH 10, P = 0.101

**Table S5 Cd content corresponding to the figure 5**

| Actual Cd content in figure 5A (mg/kg) | |
| --- | --- |
| Treatment of the lentinan | Cd content |
| 90 mg/L AC (pH 7) | 1.13a* |
| 90 mg/L PAC (pH 7) | 1.33b |
| 90 mg/L Chitosan (pH 7) | 1.44bc** |
| mixture of AC, PAC (pH 7) | 1.13ad*** |
| Actual Cd content in figure 5B (mg/kg) | |
| Treatment of the lentinan | Cd content |
| pH 5 (50 mg/L AC) | 1.54a |
| pH 6 (50 mg/L AC) | 1.34b |
| pH 7 (50 mg/L AC) | 1.13c |
| pH 8 (50 mg/L AC) | 0.95d |
| pH 9 (50 mg/L AC) | 0.89e |
| pH 10 (50 mg/L AC) | 0.82ef |
| Actual Cd content in figure 5C (mg/kg) | |
| Treatment of the lentinan | Cd content |
| 50 mg/L AC pH 9 without preoxidation | 0.80a |
| 50 mg/L AC pH 9 preoxidation | 0.51b |

* Values (means of three replicates) not sharing common letters are significantly different (one-way ANOVA with the Student−Newman−Keuls method, P < 0.05); the values of 5A, 5B or 5C were analyzed independently

** PAC vs. Chitosan, P = 0.142

*** AC vs. Mix, P =1.000

**** pH 9 vs. pH 10, P = 0.192

**Table S6 Cd content corresponding to the figure 6**

| Actual Cd content in figure 6 (mg/kg) | |
| --- | --- |
| sample | Cd content |
| Initial Cd content in the mushroom | 19.49a* |
| Initial Cd content in the lentinan | 2.77b |
| Lentinan treated by optimized process | 0.10c |

*Values (means of three replicates) not sharing common letters are significantly different (one-way ANOVA with the Student−Newman−Keuls method, P < 0.05)
